# Detection of human cytomegalovirus and Epstein-Barr Virus in symptomatic and asymptomatic apical periodontitis lesions by real-time PCR

**DOI:** 10.4317/medoral.18905

**Published:** 2013-05-31

**Authors:** Selcuk M. Ozbek, Ahmet Ozbek, Muhammed S. Yavuz

**Affiliations:** 1 Endodontist Dt Ph.D, Oral and Dental Health Center, Eskişehir, Turkey; 2 Associate Professor, Department of Microbiology and Clinical Microbiology, Medical Faculty, Sakarya University, Sakarya, Turkey; 3 Associate Professor, Department of Oral and Maxillofacial Surgery, Dental School of Atatürk University, Erzurum, Turkey

## Abstract

Objectives: Recent studies have investigated the occurrence of human cytomegalovirus and Epstein-Barr Virus in samples from apical periodontitis lesions and a role in the pathogenesis of this disease has been suggested. Because genotype distribution and seroprevalence of EBV and HCMV differ among populations, it is important to determine the presence of these viruses in endodontic periapical lesions of different populations. The aims of this study were to determine the presence of HCMV and EBV DNAs in samples from Turkish patients with symptomatic and asymptomatic apical periodontitis lesions using real-time polymerase chain reaction method and to evaluate their presence in both symptomatic and asymptomatic apical periodontitis lesions. Study Design: Periapical samples were collected from 12 asymptomatic and 16 symptomatic periapical lesions in conjunction with apicectomy. HCMV and EBV DNAs were identified in the samples by real-time PCR. The chi-squared test with Yates’s correction or the Fisher’s exact test was used to analyse the significance of differences. Results: HCMV DNA was detected in 10 of the 16 (62.5%) symptomatic and in five of the 12 (41.7 %) asymptomatic periapical study lesions. The EBV DNA was identified in seven of the 16 (43.7 %) symptomatic and three of the 12 (25 %) asymptomatic periapical lesions. The difference in occurrence of HCMV and EBV DNA between symptomatic and asymptomatic periapical lesions was not statistically significant. (All comparisons have p > 0.05). Conclusions: Our findings suggest that HCMV and EBV is a frequent inhabitant of both symptomatic and asymptomatic apical periodontitis lesions of endodontic origin in Turkish population.

** Key words:**Human cytomegalovirus, Epstein-Barr Virus, apical periodontitis, Polymerase chain reaction method.

## Introduction

Apical periodontitis is an inflammatory disease that affects the tissues surrounding the apical portion of the dental root and is primarily caused by microorganisms infecting the root canal (1). Symptomatic apical periodontitis occurs within a previously healthy periapical region in response to either microbiological or physical irritation. Teeth with symptomatic apical periodontitis will have very marked tenderness to percussion and pain when pressure is applied to the tooth. It may or may not be associated with an apical radiolucent area (2,3). Acute periapical infection eventually turns into a chronic state predominated by macrophages, lymphocytes and plasma cells encapsulated in collagenous connective tissue (1,4). Asymptomatic apical periodontitis is a long-standing periapical inflammatory lesion with radio graphically visible periapical bone resorption but with minimal or no clinical symptoms. Histopathologically, it consists of granulomatous tissue with infiltrate cells, fibroblasts, and a well-developed fibrous capsule (1,5).

In the past two decades, methods based on molecular biology have been introduced for microbial identification. The molecular methods most often used for microbial identification are the polymerase chain reaction (PCR) method and its variations (6). Most PCR assays are qualitative or can be semi quantitative. One exception is real-time PCR, which is characterized by the continuous measurement of products throughout the reaction (7). The advantages of real-time PCR are the rapidity of the assay, the ability to quantify and identify PCR products directly without the use of agarose gels (6).

The recent use of PCR based molecular methods to detect herpes viruses in periradicular lesions has suggested that some herpes-viruses, especially human cytomegalovirus (HCMV) and Epstein-Barr virus (EBV) can participate in the pathogenesis of the periradicular lesions (4,6,8-11). Slots et al. (11) hypothesized that some types of aggressive periapical pathosis develop as a result of a series of interactions among herpes viruses, bacteria, and host immune reactions. It was also suggested that cooperation between herpes viruses and endodontopathic bacteria play major roles in the etiopathogenesis of aggressive types of periapical pathosis (10).

Viruses of the family Herpesviridae are widespread in the human population (12). The prototypical structure of herpes viruses consists of a double-stranded DNA genome encased within an icosahedral capsid, a proteinaceous tegument and a lipid-containing envelope with embedded viral glycoproteins (13,14). Eight human herpes virus species with distinct biological and clinical characteristics have been described; herpes simplex virus-1, herpes simplex virus-2, varicella–zoster virus, EBV, HCMV, human herpes virus-6, human herpes virus-7 and human herpes virus-8 (14). The initial herpes virus infection is followed by a latent phase in host cells, which ensures the survival of the viral genome throughout the lifetime of infected individuals. Herpes virus reactivation may occur spontaneously or as a result of concurrent infection, fever, drugs, tissue trauma, emotional stress, and other factors impairing the host immune defense (11).

Herpes viruses express proteins during the normal lytic and latent viral life cycle that can interfere with activities of the innate and adaptive immune systems and alter the cellular environment (14). HCMV can cause serious infectious diseases and may be found in blood and in many body secretion including saliva, urine, semen and breast milk (15). HCMV infects many different epithelial cells, endothelial cells, smooth muscle cells, mesenchymal cells, hepatocytes, granulocytes and monocyte-derived macrophages and resides in the bone-marrow myeloid progenitor cells during latency (15,16). EBV is a known cause of infectious mononucleosis and almost certainly plays a role in the etiology of nasopharyngeal carcinoma, Burkitt’s lymphoma, and lymphoproliferative disorders in the presence of immunosuppression (11). This virus is usually transmitted by oral secretions or blood (15). EBV infects relatively long-lived B-lymphocytes during both primary and latent infections, and can infect the oropharyngeal epithelium (17).

The findings obtained from different studies with different sample of patients revealed a strong association of EBV and HCMV with symptomatic periapical lesions (12). Because genotype distribution and seroprevalence of EBV and HCMV differ among populations (18), it is important to determine the presence of these viruses in endodontic periapical lesions of different populations. The aims of this study were to determine the presence of HCMV and EBV DNA in samples from Turkish patients with symptomatic and asymptomatic apical periodontitis lesions using a sensitive molecular method, i.e., real-time PCR and to evaluate their presence in both symptomatic and asymptomatic apical periodontitis lesions.

## Material and Methods

-Patient selection

The examined material was selected from adult patients seeking dental care at the Department of Oral and Maxillofacial Surgery, Dental School of Atatürk University (Erzurum, Turkey). The Ethical Committee in Research of the Dental School of Atatürk University approved the study protocol, and informed consent was obtained from each of the patients.

The inclusion criteria for patients were as follows: individuals in good health (American Society of Anesthesia I or II) with no severe systemic disease requiring surgical apicoectomy because of the failure of conventional root canal therapy. The exclusion criteria were periodontally involved teeth (probing depth is >4 mm, with periodontal bone loss), vertical root fracture, or immature teeth with open apices (19).

Age, gender, clinical symptoms and signs included pain on occlusion, tenderness to percussion or palpation, swelling, the presence of periapical radiolucency (location and size), probing depth of any periodontal pockets, history of previous and present antibiotic therapy were recorded for each patient. Medical histories revealed that all patients were in good general health, had not received antibiotics 3 months before the start of the study and had no important systemic diseases, such as diabetes.

In all patients, panoramic radiography or periapical radiographs obtained by a long cone paralleling technique were used to complement the imaging diagnostic data. To assess the size of the lesion, the greatest mesiodistal and craniocaudal diameters of the lesion were measured on standard panoramic images, and lesion size was calculated as the arithmetic mean of those values.

The study included 16 patients with symptomatic apical periodontitis and 12 patients with asymptomatic apical periodontitis ( Table 1). Symptomatic lesions were characterized by acute pain, discomfort on biting, and sensitivity by percussion or palpation at the apical region of mucosa. The asymptomatic lesions revealed no signs or symptoms of acute periapical inflammation or pain at the time of study with the exception of periapical radiolucent area on radiographs (19-21).

**Table 1:**
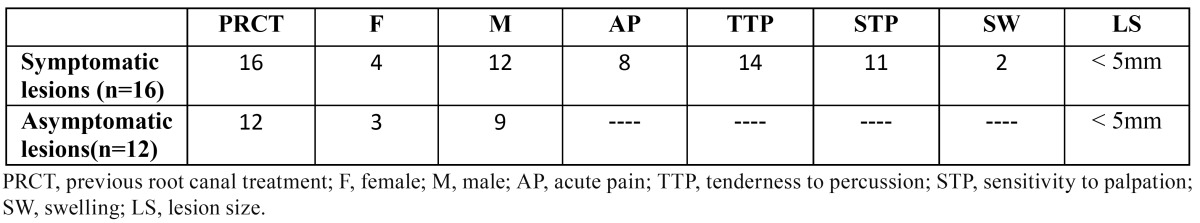
Clinical features of patients.

-Study design

Periapical samples were collected in conjunction with apicectomy, which was being performed due to radiographic evidence of incomplete periapical healing following conventional root canal treatment (a previously existing periapical lesion had increased or remained at the same size or a previously normal periodontal ligament space had increased in width or developed into radiolucent area) as described previously (4,19-21). Before administrating local anesthetics for apicoectomy, the teeth, gingiva and mucosa of the sample area were washed with 0.2% chlorhexidine and patients rinsed with 0.2% chlorhexidine mouthwash for 30s. Using a sterile No. 15 blade, gingival incisions were extended one or two teeth mesially or distally from the studied tooth followed by a vertical releasing incision. A full-thickness mucoperiosteal flap was then reflected, and the periapical lesion was exposed with a sterile round burr using sterile saline as coolant. Using a sterile curette, a periapical specimen for virological identification was placed in a sterile 2 mL Eppendorf tube as collection tube with 0.5 mL sterile distilled water and then was immediately frozen to -20ºC until DNA extraction.

-DNA extraction

The frozen samples from patients in the 2-mL collection tubes with 0.5 mL sterile distilled water were left at room temperature for 20 minutes. After the temperature of the samples had adjusted to room temperature, the samples specimens were pre-clarified by centrifugation to remove debris, prior to DNA extraction using the QIAamp® DNA mini-kit (Qiagen, Hilden, Germany). The protocol recommended by the kit manufacturer for DNA extraction from the tissue samples was followed precisely.

-PCR amplification

To amplify the DNAs of HCMV and EBV, two commercial kits Fluorion® EBV QNP 1.0 (Iontek, Istanbul, Turkiye) for EBV and Fluorion® CMV QNP 3.0 (Iontek, Istanbul, Turkiye) for HCMV used separately. The amplification and detection of DNA with virus-specific kits by real-time PCR were performed with the ICycler IQ Multicolor Real- Time PCR Detection System (Bio-Rad Laboratories, Hercules, CA, USA). The PCR amplifications with virus-specific kits were repeated one more time to confirm the results obtained during the first study.

-Data analysis

The prevalence of HCMV and EBV DNA in symptomatic and in asymptomatic apical periodontitis lesions was recorded as a percentage of the cases examined. The chi-squared test with Yates’s correction or the Fisher’s exact test was used to analyse the significance of differences. Significance levels were established at 5 % (P < 0.05).

## Results

A total of 16 symptomatic and 12 asymptomatic apical periodontitis samples were collected from 28 patients (age range, 22-36 years; average age, 29 years). The radiographic size of all the apical periodontitis lesions was less than 5 mm diameters.

Data on the prevalence of HCMV and EBV DNA in symptomatic and asymptomatic apical periodontitis lesions are presented in  Table 2. HCMV DNA was detected in 10 of the 16 (62.5%) symptomatic and in five of the 12 (41.7 %) asymptomatic periapical study lesions. The EBV DNA was identified in seven of the 16 (43.7 %) symptomatic and three of the 12 (25 %) asymptomatic periapical lesions. Clinical symptoms and the number and distribution of HCMV and EBV DNA in symptomatic apical periodontitis lesions are shown in  Table 3. The difference in occurrence of HCMV and EBV DNA between symptomatic and asymptomatic periapical lesions was not statistically significant. (All comparisons have p > 0.05). Both HCMV and EBV DNA was detected in four (25 %) symptomatic and in two (16.7 %) asymptomatic periapical samples.

**Table 2:**
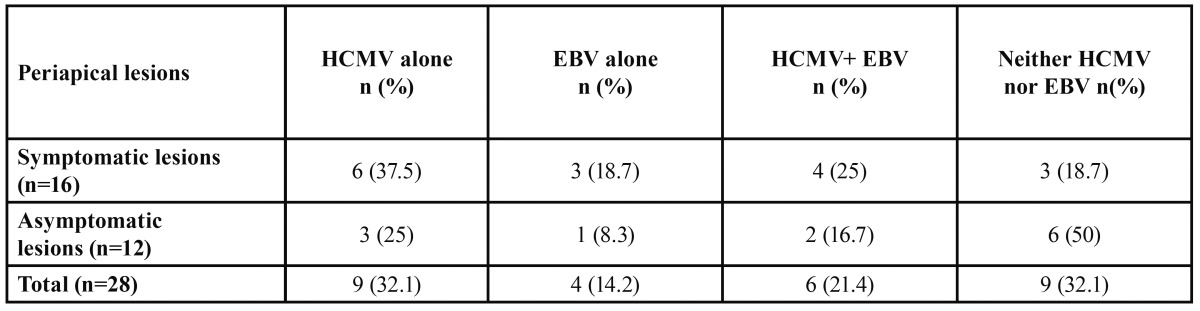
Prevalence of HCMV and EBV DNA in symptomatic and asymptomatic apical periodontitis lesions.

**Table 3:**
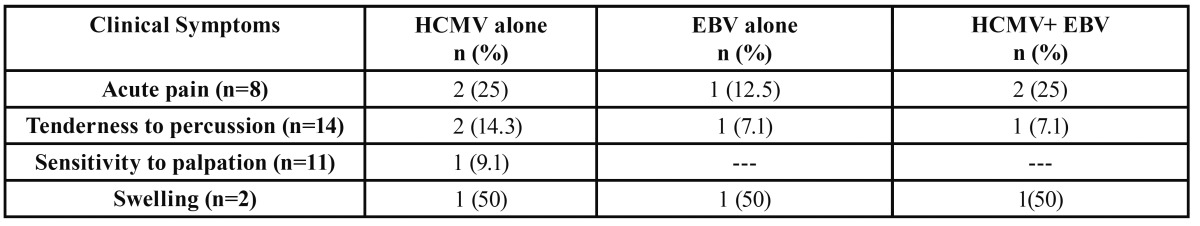
Clinical symptoms and the number and distribution of HCMV and EBV DNA in symptomatic apical periodontitis lesions.

## Discussion

There is consensus that apical periodontitis occurring after root canal treatment presents a more complex aetiological and therapeutic situation than apical periodontitis affecting teeth that have not undergone endodontic treatment. It is generally acknowledged that most failures occur when treatment procedures have not reached a satisfactory standard for the control and elimination of infection (22). Although bacteria are by far the most common microorganisms involved in endodontic infections, studies have revealed a possible role for fungi and more recently for viruses (6). Sabeti et al. (4) using RT-PCR, identified HCMV and EBV transcripts in periapical granulomatous tissues and detected that herpes viruses in large symptomatic periapical lesions at a higher incidence when compared with small asymptomatic periapical lesions. Yildirim et al. (23) demonstrated the presence of herpes viruses and bone resorption-inducing cytokines in periapical lesions of deciduous teeth. Using an immunohistochemical approach, Saboia-Dantas et al. (24) identified HCMV and EBV in apical periodontitis lesions, with higher prevalence in HIV-positive patients. Chen et al. (16) using primary and nested PCR, found HCMV and EBV in patients with acute apical abscesses and cellulitis of endodontic origin. Using PCR, Li et al. (25) identified EBV DNA and RNA in endodontic pathoses in significantly higher percentages compared with healthy pulp controls.

Herpes viruses may cause disease as a direct result of viral infection and replication, or as a result of virally induced impairment of the host defense (26). Herpes virus-mediated endodontopathogenicity may take place via several mechanisms, operating alone or in combination, and may involve both cellular and humoral host responses (4). Herpes viruses may cause direct cytopathic effects on periapical fibroblasts, endothelial cells and bone cells, the results of which may be impaired tissue turnover and repair, and ultimately loss of tissue (12,26). HCMV infection induces pro-inflammatory cytokine responses in inflammatory cells, with production of interleukin (IL)-1β, IL-6, IL-12, tumour necrosis factor (TNF)-α, interferon (IFN)-α/β, IFN-γ and prostaglandin E2 (PGE2). Cytokines and chemokines produced in EBV infection include IL-1β, IL-1receptor antagonist (IL-1Ra), IL-6, IL-8, IL-18, TNF-α, IFN- α/β, IFN-γ, monokine induced by IFN-γ, IFN-γ -inducible protein10 (IP-10) and granulocyte-macrophage colony-stimulating factor (9,20,27). These inflammatory mediators, which are most likely produced locally by periapical macrophages, are potent bone resorption-stimulating agents (4,12,28). Herpes virus-induced immune impairment may also cause an upgrowth of resident Gram-negative anaerobic bacteria (10) whose lipopolysaccharide can induce cytokine and chemokine release from various mammalian cells and may act synergistically with HCMV in stimulating IL-1b gene transcription (20,29).

Herpes viral-bacterial interactions may help explain various clinical characteristics of periapical infections (11). Alternation between prolonged periods of virus latency interrupted by periods of activation may be hypothesized as being partly responsible for the intermittent symptoms of apical periodontitis (24). The cumulative effects of herpes viruses, endopathogenic bacteria, and proinflammatory immune mechanisms may be manifested in increased resorption of the periapical alveolar bone and in clinical symptoms, such as acute pain, discomfort on biting, and sensitivity to palpation at the apical region of mucosa (19).

The real-time PCR method used in the present study is a powerful technique, with the advantages of rapidity of the assay, ability to quantify and identify PCR products directly without the use of agarose gels, and the fact that contamination of the nucleic acids is limited because of avoidance of post amplification manipulation (6,7). In the present study, HCMV and EBV DNA were detected in 53.6% (15/28) and in 35.7% (10/28), respectively, of the all samples taken from periapical lesions by real-time PCR. Sabeti et al. (4) using primary PCR, identified HCMV transcript in almost all periapical specimens from patients previously treated with apical periodontitis. Using PCR, Slots et al. (20) found HCMV in all symptomatic and in 37% of the asymptomatic study lesions and they identified EBV only in HCMV infected periapical lesions. These results are in contrast to this study in which much lower incidence of HCMV DNA (in 62.5% of symptomatic and in 41.7% of asymptomatic) are found in samples from previously treated with apical periodontitis. In addition, we also found higher incidence of HCMV DNA from periapical samples compared findings of Li et al. (25) and Sunde et al. (30). Using primary, nested and reverse transcription PCR, Li et al. (25) found HCMV DNA in 4.4% of the samples with previously treated with apical periodontitis. Sunde et al. (30) found EBV in 45% of the apical periodontitis samples but did not find HCMV in their specimens by means of real-time PCR. The differences may occur several reasons including differences in the methods used for DNA extraction, several host and environmental factors such as genetic background, ethnicity and socioeconomic status (17,18). Results from this study are more consistent with the findings of Yazdi et al. (21), Saboia-Dantas et al. (24). Using PCR, Yazdi et al. (21) have found HCMV to be the predominant herpes virus (42% incidence) in previously treated with apical periodontitis samples compared with EBV which was detected in 6% of their samples. Saboia-Dantas et al. (24) have identified HCMV and EBV in 23% and 31% of patients with apical periodontitis, respectively.

The detection of herpes virus DNA in periapical lesions has brought a new dimension to our knowledge of periapical infections (20). Whether active herpesvirus infections in periapical sites initiate or contribute to disease flare-up, or whether herpesvirus activation occurs secondarily to acute inflammation remains an important research topic (9). In conclusion, the present PCR based findings have identified HCMV and EBV as a frequent inhabitant of apical periodontitis lesions of endodontic origin in Turkish population. Additional studies using animal models are required to elucidate the role of herpes viruses in the pathogenesis of periapical pathosis.
